# Case report: Corticosteroids-induced acute diabetic peripheral neuropathy

**DOI:** 10.3389/fendo.2022.914325

**Published:** 2022-08-03

**Authors:** Jia-Lin Yuan, Le Sun, Bao-Lin Su, Chuang-Xiong Hong

**Affiliations:** ^1^ Cardiovascular Department, Guangzhou University of Chinese Medicine, Guangzhou, China; ^2^ The Department of Nephrology, The First Affiliated Hospital of Guangzhou University of Chinese Medicine, Guangzhou, China; ^3^ The Department of Cardiovascular Disease, The First Affiliated Hospital of Guangzhou University of Chinese Medicine, Guangzhou, China

**Keywords:** corticosteroids, IgA nephropathy, diabetes mellitus, acute diabetic peripheral neuropathy, adverse drug reaction, diabetic lumbosacral radiculoplexus neuropathy

## Abstract

A 62-year-old man was diagnosed as IgA nephropathy. He had a pancreatic tumor operation 19 years ago and had a normal plasma glucose test every year. One month after the medication of prednisolone acetate was administered his fasting plasma glucose elevated to 7.1mmol/L while he manifested symptoms of thirst, frequent urination, and weight loss. Approximately 3 months after the steroids, he started complaining of numbness, weakness, and muscle cramp in his lower extremities, blood tests showed elevated plasma glucose and electromyography (EMG) revealed impairment of the peripheral nerves in the lower extremity, diabetic peripheral neuropathy was diagnosed. Mecobalamin and Acupuncture were employed and steroids were discontinued, 8 months later he recovered part of his strength and sensation. This case presents a specific adverse drug reaction of corticosteroids that causes diabetes mellitus and subsequently leads to peripheral neuropathy in an acute onset.

## Introduction

Diabetic peripheral neuropathy (DPN) is the presence of symptoms and/or signs of peripheral nerve dysfunction in people with diabetes, after exclusion of other causes (1). DPN could be caused by type 1 or type 2 diabetes mellitus (DM). Its incidence among diabetic patients is high, and it is usually seen in those who have a long history of DM, with a close relationship to poor glycemic control. The onset of DPN can be acute and sudden, like diabetic lumbosacral radiculoplexus neuropathy (DLRPN) and insulin neuritis. But DPN is more common in the form of chronic and length-dependent complications of DM, such as distal symmetric polyneuropathy (DSPN) (2).

It is also known that corticosteroids elevate blood glucose and causes iatrogenic or steroids-related DM, yet it is rare that DPN happens in steroids-related DM patients, much less in an acute onset. Here we present a case with IgA nephropathy manifested clinical symptoms of DM and developed DPN after he received medication of corticosteroids in less than 3 months.

## Case report

A 62-year-old man with a history of pancreatic tumor operation was diagnosed with IgA nephropathy through renal biopsy. Denying previous use of corticosteroids or chronic intake of alcohol and having a normal result of plasma glucose level and fundus examination, he started receiving medication of prednisolone acetate in a dose of 30mg PO QD immediately after discharge. One month later his blood test showed fasting blood glucose (FBG) elevated to 7.1mmol/L, this test result was unnoticed. Subsequently, the dose of prednisolone acetate was reduced to 25mg QD because of the reduction of 24-hour urinary protein quantity, at this period he gradually manifested increased thirst, frequent urination, and unexplained weight loss. Two months later a routine blood test revealed FBG was 17mmol/L, 2-hour postprandial blood glucose (2hPG) 27.87mmol/L, HbA1c 8.0%. He was unchecked until he complained of symptoms of numbness, weakness, and paresthesia pain in his left lateral crural region and dorsum pedis, with transient pain and muscle cramp in his bilateral thighs, this happened nearly 3 months after the first medication of prednisolone acetate.

He was admitted to the hospital with normal vital signs; blood tests revealed his postprandial plasma glucose 27.98mmol/L, HbA1c 12.2%, ketone body 1.4mmol/L, lactate 2.27mmol/L, ionized calcium 1.08mmol/L, pH 7.434, standard bicarbonate 27.2mmol/L, actual bicarbonate 27.6mmol/L, albumin 32.6g/L, serum creatinine 101.4 μmol/L, c-reactive protein, and erythrocyte sedimentation rate was normal. Serum antibodies testes showed C3 0.620g/L, C4 0.182g/L, antibodies against cyclic citrullinated peptides (anti-CCP), antibodies of antinuclear antibody (ANA) profile and antineutrophil cytoplasmic antibodies (ANCA) all returned negative. The first EMG showed impairment of the left common peroneal nerve and superficial peroneal nerve(see Table 1), and in needle EMG examination of the tibialis anterior, the patient failed to complete the maximum contraction test. Color Doppler sonography indicated bloodstream of bilateral arteries and veins was normal though arteriosclerosis was present. Unfortunately, neurological physical examination was not well-recorded due to the focus on nephropathy and diabetes mellitus. Diagnosis of DM and peripheral neuropathy was confirmed and steroids were stopped, followed by administration of insulin and mecobalamin, acupuncture therapy was also employed.

Four months later he was re-admitted to the hospital, though he still complained of continuous numbness and weakness in his left lateral crural region and dorsum pedis, muscle cramp and pain in the bilateral thighs had ameliorated. His weight reduced from 72 kg to 61 kg in a month, a blood test showed HbA1c 8.1%, physical examination revealed mild amyotrophy in the bilateral lower leg and the left one was more severe. Excessive ankle plantar flexion (drop foot) of the left foot and slight pitting edema in the bilateral ankle was also observed. The strength of dorsiflexion in the left foot was grade 1/5, with numbness in the region of the left lateral crural and dorsum pedis, and the left knee reflex was (+). A re-checked EMG results indicated impairment of the bilateral sural nerve, bilateral superficial peroneal nerve and left common peroneal nerve(see Table 2), in accordance with the results of physical examination. We did an MRI scan in the patient’s lumbar spine and found mild lumbar disc herniation at L4-L5 and L5-S1, but the Lasegue sign and Bragard sign was negative in both legs. After consulting the orthopedist, the lumbar disc herniation was considered irrelevant to the symptoms.

After excluding other possibilities, we confirmed the diagnosis of DPN, which was induced by corticosteroids. Medication of mecobalamin and acupuncture therapy had been continued for DPN and 8 months later in the follow-up visit, the strength of dorsiflexion of the left foot was partly recovered and he was capable of running again.

## Discussion

DM has become a global problem, with 27% of individuals older than 65 years having DM, and the effects of hyperglycemia on the neuromuscular system are well-known and hard to handle, and it is estimated that diabetic neuropathy (DN) affects approximately 50% of DM patients (3, 4). DN is a difficult clinical issue, it often takes months or even years to control. DN encompasses distal symmetric polyneuropathy (DSPN), diabetic autonomic neuropathies, mononeuropathy, and radiculopathy/polyradiculopathy (5). In this case, the onset of DM induced by corticosteroids is 8 weeks after the intake of prednisolone acetate, and conditions of peripheral neuropathy started approximately 11~12 weeks after the corticosteroids, i.e., 3 weeks after symptoms of DM appeared.

We tend to believe the presented case is caused by diabetic polyradiculopathy or more exactly, diabetic lumbosacral radiculoplexus neuropathy (DLRPN), which involves the lumbosacral plexus, and occurs in men with type 2 diabetes mellitus, characterized by acute or subacute onset, extreme unilateral thigh pain, weight loss, and subsequent motor weakness (5, 6). It is a rare complication associated with early-stage diabetes and is related to the ischemic injury caused by inflammatory microvasculitis (7, 8). The median time from diagnosis of diabetes mellitus to onset of disease was 4.1 years (9). Diagnosis could be made through electrophysiologic abnormalities (6). Our patient developed symptoms of DLRPN 3 weeks after the manifestation of DM appeared, starting with bilateral transient thigh pain, numbness, weakness, and paresthesia pain in his left lateral crural region and dorsum pedis, physical examination revealed mild amyotrophy in the bilateral lower leg and excessive ankle plantar flexion. These results are congruent with the impairment of the left common peroneal nerve. Originating from the sciatic nerve, the common peroneal nerve controls muscles including tibialis anterior, extensor digitorum longus, extensor hallucis longus, peroneus longus, and peroneus brevis, with the sensory fibers mainly located in the anterior, lateral area of the lower leg and dorsum pedis. The damage to the common peroneal nerve presents with atrophy of the calf extensor muscle, drop foot (excessive ankle plantar flexion), incapability of dorsiflexion, and abnormal sensation in the anterior, lateral skin of the lower leg and dorsum pedis. Both EMG results confirmed the damage to the common peroneal nerve (see Table 1), which could be also validated by the needle EMG examination in the test of tibialis anterior.

Thus, we suggest the diagnosis of DLRPN, which was caused by diabetes mellitus induced by corticosteroids. We also made differential diagnoses of several similar diseases. The first to be concerned about is Guillain-Barre Syndrome (GBS), which is an acute inflammatory demyelinating polyneuropathy that manifests progressive and symmetrical weakness and abnormal sensation in the extremities, which in most cases of GBS ceases progression within 4 weeks (10). In our case, the weakness happened in an acute onset and reached the highest level in a few days, with unsymmetrical symptoms and partial nerve damage not inflicting the entire leg, as the patient had normal strength of plantarflexion, knee extension, and knee flexion in the left foot. EMG also showed a significant decrease in nerve amplitude and M-wave amplitude in the left common peroneal nerve and the superficial peroneal nerve, with a reduction of conduction velocity that did not exceed 75% of the normal range in the left common peroneal nerve, indicating possible axon damage or axon degeneration, rather than demyelination which presents in GBS that is characterized by an increase of latency, and a significant decrease of conduction velocity. Since this case is a middle-aged man, an atypical type of GBS is not considered (11).

It is noteworthy that the patient suffered from pancreatic tail carcinoma (non-functional islet cell carcinoma) in 2003 and the tumor was successfully removed. He experienced chemotherapy 12 times after the surgery. So far his routine screenings for cancer have not seen any sign of recurrence, therefore the tumor was considered irrelevant to the conditions. Amyloidosis induced by IgA nephropathy was considered most unlikely due to the infliction of peripheral nerve in the lower extremities while amyloidosis could also present in the heart, liver, and kidney (12). It has a long duration, difficult to ameliorate, and may aggravate proteinuria, so it is the last to be considered. Because of the incapacity of testing Vitamin B1, B6, B12, E, or copper in our hospital, we did not investigate further. In a word, we think this is a case diagnosed as DPN induced by corticosteroids with an acute onset.

To the best of our knowledge, this is the first report of DPN caused by corticosteroid-induced DM. Literature review showed that corticosteroids are the first-line therapy of DPRPN and the majority of them achieved great efficacy (13, 14). Double-blinded, randomized control trial also validated the efficacy of corticosteroids in the pain management of DLRPN (15). However, the history of our case suggested that corticosteroids may have the potential to indirectly cause DLRPN. It is assumed that the acute development of DM and DLRPN may be relevant to the history of pancreatic tumor operation 19 years ago. Despite his plasma glucose level being normal before the steroid medication, the operation had removed most of his pancreas and the balance of blood glucose level had been sustained in a delicate manner.

In the latest follow-up, the patient had a good recovery in strength, and symptoms of numbness had been ameliorated greatly. In his words, he estimated “80% recovery”. The swift amelioration surprised us and it could be boiled down to the prompt withdrawal of the corticosteroids and undertaking of appropriate treatment.

In conclusion, we present the first case of DPN induced by corticosteroids, we also suggest the use of corticosteroids be considered as a risk factor for DPN and further research is needed. Still, caution should be raised for health practitioners during the administration of corticosteroids and measurements should be taken when symptoms of peripheral neuropathy develop after taking medication of corticosteroids in case of further deterioration.

## Data availability statement

The original contributions presented in the study are included in the article/**Supplementary Material**. Further inquiries can be directed to the corresponding author.

## Ethics statement

The studies involving human participants were reviewed and approved by ethics committee of The First Affiliated Hospital of Guangzhou University of Chinese Medicine. The patients/participants provided their written informed consent to participate in this study.

## Author contributions

All authors listed have made a substantial, direct, and intellectual contribution to the work and approved it for publication.

## Conflict of interest

The authors declare that the research was conducted in the absence of any commercial or financial relationships that could be construed as a potential conflict of interest.

## Publisher’s note

All claims expressed in this article are solely those of the authors and do not necessarily represent those of their affiliated organizations, or those of the publisher, the editors and the reviewers. Any product that may be evaluated in this article, or claim that may be made by its manufacturer, is not guaranteed or endorsed by the publisher.
