# Supplementary material for: Case report: Corticosteroids-induced acute diabetic peripheral neuropathy
Source: Front Endocrinol (Lausanne). 2022 Aug 3;13:914325. doi: 10.3389/fendo.2022.914325 (PMC9381879; doi:10.3389/fendo.2022.914325)
Supplement: Supplementary file 1 [file Table_1.docx]

| Motor Studies  Nerve | Segment | | | Latency  (ms) | | Amplitude  (mV) | | Distance  (mm) | Conduction Velocity  (m/s) |
| --- | --- | --- | --- | --- | --- | --- | --- | --- | --- |
| Tibial.R |  | | | | | | | | |
| Ankle | Extensor digitorum brevis-Ankle | | | 4.7 | | 11.7 | |  |  |
| Fibula(head) | Ankle-Fibula(head) | | | 13.2 | | 14.4 | | 380 | 45 |
|  | | | | | | | | | |
| Tibial.L |  | | | | | | | | |
| Ankle | Extensor digitorum brevis-Ankle | | | 4.5 | | 13.2 | |  |  |
| Fibula(head) | Ankle-Fibula(head) | | | 12.7 | | 10.7 | | 375 | 46 |
|  | | | | | | | | | |
| Peroneal. R |  | | | | | | | | |
| Fibula(head) | Tibialis anterior-Fibula(head) | | | 3.9 | | 5.1 | |  |  |
| Popliteal fossa | Fibula(head)-Popliteal fossa | | | 12.3 | | 2.9 | | 360 | 43 |
|  | | | | | | | | | |
| Peroneal. L |  | | | | | | | | |
| Fibula(head) | Tibialis anterior-Fibula(head) | | | 3.2 | | 1.1 | |  |  |
| Popliteal fossa | Fibula(head)-Popliteal fossa | | | 12.2 | | 0.3 | | 345 | 38 |
| Sensory Studies  Nerve | | Segment | Onset Latency  (ms) | | Peak Latency  (ms) | | Amplitude  (μV) | Distance  (mm) | Conduction Velocity  (m/s) |
| Sural. R | |  | | | | | | | |
| Ankle | | Lower Leg-Ankle | 2.2 | | 3.8 | | 19 | 140 | 64 |
|  | | | | | | | | | |
| Sural. L | |  | | | | | | | |
| Ankle | | Lower Leg-Ankle | 2.1 | | 4.0 | | 15 | 135 | 64 |
|  | | | | | | | | | |
| Superficial Peroneal. R | |  | | | | | | | |
| Ankle | | Dorsum of foot-Ankle | 2.3 | | 4.5 | | 16 | 130 | 57 |
|  | | | | | | | | | |
| Superficial Peroneal. L | |  | | | | | | | |
| Ankle | | Dorsum of foot-Ankle | 2.6 | | 5.0 | | 7 | 155 | 59 |

| F-Wave Studies  Nerve | M-Wave Latency | F-Wave Latency | F-Wave Occurence | F-Wave Conduction Velocity |
| --- | --- | --- | --- | --- |
| Tibial. R | 5.1 | 48.1 | 80% |  |
| Tibial. L | 5.1 | 48.8 | 100% |  |

| H-Wave Studies  Nerve | M-Wave Latency  (ms) | H-Wave Latency  (ms) | M-Wave Amplitude(Max)  (mV) | H-Wave Amplitude(Max)  (mV) |
| --- | --- | --- | --- | --- |
| Tibial. R | 3.7 | 30.8 | 2.0 | 0.4 |
| Tibial. L | 4.4 | 30.8 | 0.8 | 0.1 |
