# Supplementary material for: Case report: Corticosteroids-induced acute diabetic peripheral neuropathy
Source: Front Endocrinol (Lausanne). 2022 Aug 3;13:914325. doi: 10.3389/fendo.2022.914325 (PMC9381879; doi:10.3389/fendo.2022.914325)
Supplement: Supplementary file 2 [file Table_2.docx]

| Motor Studies  Nerve | Segment | | Latency  (ms) | Amplitude  (mV) | Distance  (mm) | Conduction Velocity  (m/s) |
| --- | --- | --- | --- | --- | --- | --- |
| Tibial. R |  | | | | | |
| Ankle | Abductor hallucis-Ankle | | 5.7 | 7.2 |  |  |
| Popliteal fossa | Ankle-Popliteal fossa | | 13.8 | 7.2 | 365 | 45 |
|  | | | | | | |
| Tibial. L |  | | | | | |
| Ankle | Abductor hallucis-Ankle | | 4.4 | 8.5 |  |  |
| Popliteal fossa | Ankle-Popliteal fossa | | 13.4 | 8.3 | 380 | 42 |
|  | | | | | | |
| Peroneal. R |  | | | | | |
| Ankle | Extensor digitorum brevis-Ankle | | 3.2 | 2.1 |  |  |
| Fibula(head) | Ankle-Fibula(head) | | 10.7 | 2.0 | 315 | 42 |
|  | | | | | | |
| Peroneal. L |  | | | | | |
| Ankle | Extensor digitorum brevis-Ankle | | 3.3 | 1.9↓ |  |  |
| Fibula(head) | Ankle-Fibula(head) | | 10.3 | 2.0 | 295 | 42 |
| Sensory Studies  Nerve | | Segment | Latency  (ms) | Amplitude  (μV) | Distance  (mm) | Conduction Velocity  (m/s) |
| Sural. R | |  | | | | |
| Lower Leg | | Ankle-Lower Leg | 2.8 | 7.0↓ | 130 | 46 |
|  | | | | | | |
| Sural. L | |  | | | | |
| Lower Leg | | Ankle-Lower Leg | 2.7 | 9.4↓ | 120 | 44 |
|  | | | | | | |
| Superficial Peroneal. R | |  | | | | |
| Ankle | | Dorsum of foot-Ankle | 2.9 | 6.9↓ | 140 | 48 |
|  | | | | | | |
| Superficial Peroneal. L | |  | | | | |
| Ankle | | Dorsum of foot-Ankle | 2.9 | 8.9↓ | 150 | 52 |

| F-Wave Studies  Nerve | M-Wave Latency | F-Wave Latency | F-Wave Occurence | F-Wave Conduction Velocity |
| --- | --- | --- | --- | --- |
| Tibial. R | 5.8 | 51.3 | 100% | 42.7 |
| Tibial. L | 4.4 | 50.6 | 100% | 42.0 |
